# The Combined Human Genotype of Truncating *TTN* and *RBM20* Mutations Is Associated with Severe and Early Onset of Dilated Cardiomyopathy

**DOI:** 10.3390/genes12060883

**Published:** 2021-06-08

**Authors:** Anna Gaertner, Julia Bloebaum, Andreas Brodehl, Baerbel Klauke, Katharina Sielemann, Astrid Kassner, Henrik Fox, Michiel Morshuis, Jens Tiesmeier, Uwe Schulz, Ralph Knoell, Jan Gummert, Hendrik Milting

**Affiliations:** 1 Herz-und Diabeteszentrum NRW, Universitätsklinikum der Ruhr-Universität Bochum, Klinik für Thorax- und Kardiovaskularchirurgie, Georgstr. 11, D-32545 Bad Oeynhausen, Germany; agaertner@hdz-nrw.de (A.G.); Julia.Bloebaum@gmx.de (J.B.); abrodehl@hdz-nrw.de (A.B.); bklauke@hdz-nrw.de (B.K.); katharina.frey@uni-bielefeld.de (K.S.); akassner@hdz-nrw.de (A.K.); hfox@hdz-nrw.de (H.F.); mmorshuis@hdz-nrw.de (M.M.); jens.tiesmeier@muehlenkreiskliniken.de (J.T.); Uwe.Schulz@helios-gesundheit.de (U.S.); jgummert@hdz-nrw.de (J.G.); 2 Erich und Hanna Klessmann-Institut für Kardiovaskuläre Forschung und Entwicklung, Georgstr. 11, D-32545 Bad Oeynhausen, Germany; 3 Bioscience, Cardiovascular, Renal & Metabolism, BioPharmaceuticals R&D, AstraZeneca, SE-431 50 Gothenburg, Sweden; ralph.knoell@ki.se; 4 Department of Medicine (MedH), Integrated Cardio Metabolic Centre (ICMC), Heart and Vascular Theme, Karolinska Institute, SE-171 77 Stockholm, Sweden

**Keywords:** cardiomyopathy, mutation, *RBM20*, TTN, haploinsufficiency

## Abstract

A major cause of heart failure is cardiomyopathies, with dilated cardiomyopathy (DCM) as the most common form. Over 40 genes are linked to DCM, among them *TTN* and *RBM20*. Next Generation Sequencing in clinical DCM cohorts revealed truncating variants in *TTN* (*TTN*tv), accounting for up to 25% of familial DCM cases. Mutations in the cardiac splicing factor RNA binding motif protein 20 (*RBM20*) are also known to be associated with severe cardiomyopathies. *TTN* is one of the major *RBM20* splicing targets. Most of the pathogenic *RBM20* mutations are localized in the highly conserved arginine serine rich domain (RS), leading to a cytoplasmic mislocalization of mutant *RBM20*. Here, we present a patient with an early onset DCM carrying a combination of (likely) pathogenic *TTN* and *RBM20* mutations. We show that the splicing of *RBM20* target genes is affected in the mutation carrier. Furthermore, we reveal *RBM20* haploinsufficiency presumably caused by the frameshift mutation in *RBM20*.

## 1. Introduction

Dilated cardiomyopathy (DCM) is one of the most common causes of heart failure (HF), with an estimated prevalence of 1 in 200–500 people and the most common indication for heart transplantation (HTx) [1,2,3]. DCM is defined by left-ventricular chamber dilatation in combination with systolic dysfunction [4]. More than 40 genes are linked to the etiology of DCM [5,6].

*RBM20* is a cardiomyopathy-associated gene (MIM #613172), which is predominantly expressed in striated muscle with highest expression in the heart [7,8,9,10]. *RBM20*-associated DCM is highly penetrant and clinically aggressive. Furthermore, it is characterized by an early onset and an increased risk for malignant ventricular arrhythmias [11,12]. RNA binding motif protein 20 (*RBM20*) belongs to the serine and arginine rich (SR) like proteins. It contains domains, which are also characteristic for other splicing factors like the ribonucleic acid recognition motif (RRM) and an RS domain, which are highly conserved between orthologues and are essential for nuclear retention of *RBM20* [13,14,15]. As a major splicing regulator of the *TTN*-transcripts, *RBM20* influences the myocardial isoform composition of the giant sarcomeric protein titin [8,13]. The *RBM20*-dependent aberrant splicing of *TTN* contributes to the DCM phenotype of *RBM20* mutation carriers. *RBM20* controls tissue-specific isoform expression of several other cardiac genes, including genes encoding Ca^2+^- and ion-handling proteins such as *CAMK2D* and *RYR2* [8,13]. Parikh et al. identified two regions in *RBM20* (in exons 9 and 11) which are genetic hotspots for cardiomyopathy associated mutations [12]. Most of the pathogenic *RBM20* mutations are localized in the highly conserved RS domain of the protein [16]. Other cardiomyopathy associated mutations were found in the Glu-rich region of the protein [10,16]. Although there are several reports of nonsense or frameshift variants in *RBM20* associated with DCM or left ventricular non-compaction cardiomyopathy (LVNC), the pathomechanism of these mutations remains unclear [17,18,19,20,21,22,23,24,25].

*TTN* encodes the giant sarcomere protein titin which plays important roles in structure and function of cardiac and skeletal sarcomeres [26,27]. *TTN* is established as a DCM-associated gene [28,29,30], with truncating variants in *TTN* (*TTN*tvs) being responsible for 15–25% of genetic DCM cases [31,32,33].

In this study, we identified, in an index patient with DCM and the need for the implantation of a total artificial heart (TAH), a missense mutation and a mutation leading to a preliminary stop codon in the genes *RBM20* (p.Gly603Arg and p.Glu792GlyfsTer9) as well as in *TTN* (p.Glu8271Gln and p.Lys23669Ter). The patient showed aberrant myocardial splicing of *TTN* and *RYR2*. *TTN* and *RYR2* splicing were normal in the patient’s father and cousin who also had DCM but carried only the *TTN* mutations. Quantitative real-time polymerase chain reaction (qRT-PCR) revealed reduced *RBM20* mRNA expression in the explanted myocardium of the index patient and RNA-sequencing showed a reduced frequency of the mutant allele. Here, we report for the first time that a frameshift mutation in *RBM20* leads to *RBM20* haploinsufficiency resulting in consequence in a splicing deficiency of its splicing targets. Furthermore, we show that a combination of (likely) pathogenic *TTN* and *RBM20* mutations leads to a severe cardiac phenotype. 

## 2. Materials and Methods

### 2.1. Clinical Description of the Patients

All probands underwent comprehensive cardiac examinations at the Heart and Diabetes Centre NRW (Bad Oeynhausen, Germany), including 12-lead electrocardiogram, echocardiography, and a coronary angiogram if indicated. Diagnosis of DCM was based on previously described diagnostic criteria [34]. 

**Figure 1:**
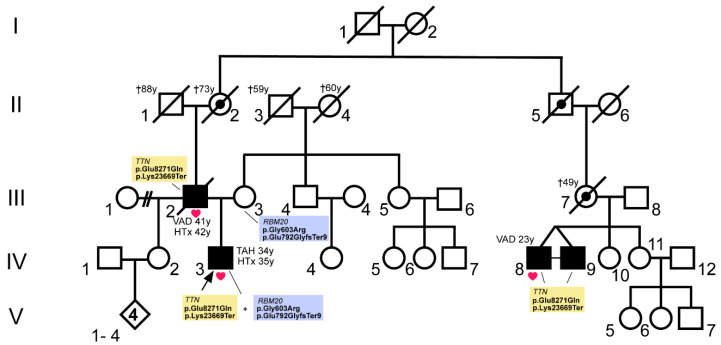
Pedigree of a cardiomyopathy family with (likely) pathogenic *RBM20* and *TTN* mutations. Circles represent females, squares males, slash denotes deceased. The index patient (IV.3) is marked with an arrow. Available myocardial tissue of patients with end-stage heart failure, who received heart transplantation (HTx) or mechanical circulatory support (left ventricular assist device (VAD) or total artificial heart (TAH)) were indicated with red heart symbols. Affected members with dilated cardiomyopathy (DCM) are shown in black and obligate mutation carriers of the *TTN* mutations are marked with a dot. The age of the patients in years (y) at the time of HTx, VAD- or TAH implantation or at the time of death (†) is specified. The respective genotypes of the analyzed patients are shown in the figure.

Index patient IV.3 (Figure 1) received his diagnosis of DCM at the age of 27 years. He was listed for heart transplantation (HTx) with 34 years and received a total artificial heart (TAH) at the same age. Orthotopic HTx was performed at the age of 35 years. The patient’s father (III.2, Figure 1) was also diagnosed with DCM and received a left ventricular assist device (LVAD) with 41 years. HTx was performed with 42 years. The patient’s mother (III.3) has arterial hypertension and showed intermittent atrial fibrillation. She has signs of a septal hypertrophy but fulfills no further cardiomyopathy criteria. The cousins of the index patient (IV.8 and IV.9, Figure 1) were diagnosed with DCM. IV.8 received an extracorporeal membrane oxygenation (ECMO) and a LVAD at the age of 23 years. Both monozygotic twins had a history of drug abuse (amphetamines, tetrahydrocannabinol).

For an overview on the clinical baseline characteristics see Table S1.

### 2.2. Genetic Analyses

Molecular genetic analyses were performed after oral and written informed consent. The local ethics committee of the Ruhr-University Bochum (Bad Oeynhausen) approved the study protocol (Reg.-No. 2018-330). The index patient (IV.3) and his cousin (IV.8) were screened for variants in 174 genes by panel sequencing using the TruSight™ Cardio gene panel (Illumina, San Diego, CA, USA) as previously described [10]. Briefly, DNA was isolated from blood using standard techniques (High Pure PCR Template Preparation Kit^®^, Roche Diagnostics GmbH, Mannheim, Germany) and prepared for cardiac gene enrichment re-sequencing on a MiSeq^®^ next generation sequencing system according to manufacturer’s instructions (TruSight™ Rapid Capture Sample Preparation Kit, Illumina). VariantStudio™ v3.0 (Illumina) was used for variant annotation. Variants of interest were verified by Sanger sequencing (BigDye^®^ Terminator v1.1 Cycle Sequencing Kit, ABI PRISM^®^ 3500 genetic analyzer, Applied Biosystems, Foster City, CA, USA). The parents of the index patient (III.2 and III.3) were genetically screened for variants found in IV.3 by Sanger sequencing. Variant classification followed the guidelines of the American College for Medical Genetics and Genomics (ACMG) [35].

### 2.3. Preparation of Myocardial Tissue

Myocardial tissue samples from the left ventricle (Biobank of the Heart and Diabetes Center NRW, Bad Oeynhausen, approved by the ethics committee of the Ruhr-University Bochum, registry No. 21/2013) were gained from the probands’ explanted heart or during implantation of a LVAD. Samples were immediately snap-frozen in liquid nitrogen and stored at −80 °C. Written consent for using their explanted myocardial tissue for research and to publish these data in anonymous form was given by all patients. This study conforms to the principles outlined in the Declaration of Helsinki [36].

### 2.4. Isolation of Total RNA

Total RNA was isolated from 30 mg of left ventricular myocardium using a commercial kit (RNeasy, Qiagen, Hilden, Germany) as previously reported [37]. Purity and RNA integrity were verified by agarose gel electrophoresis.

### 2.5. Quantitative Real Time Polymerase Chain Reaction 

Reverse transcription of myocardial RNA was performed as described previously [10], with 250 ng of total RNA and 50 units of the enzyme Superscript II (ThermoFisher Scientific, Waltham, MA, USA). For relative quantification of *RBM20*, *TTN*, and *RYR2* mRNA 2 µL of the reverse transcription reaction was used. As a housekeeping gene, *HPRT1* was used [38]. The measurements were performed in quintuplicates at the StepOnePlus™ real-time PCR system (ThermoFisher Scientific, Waltham, MA, USA). For relative quantification, the comparative cycle threshold method (ΔΔCT) of the StepOneTM software (v2.0, ThermoFisher Scientific) was used [39]. Primer sequences and PCR conditions were previously described [10]. The *RYR2* splice variant ratio corresponds to the ratio of the *RYR2* splice variant with an additional 24 bp exon against the regular *RYR2* splice variant. The *TTN* splice variant ratio corresponds to the ratio of *TTN*-N2B-splice variant to total *TTN*. 

### 2.6. Plasmid Construction

Human *RBM20* wildtype cDNA (c.385-3684) was cloned in-frame with an enhanced yellow fluorescent protein tag into the plasmid pEYFP-N1 (Takara Bio, Mountain View, CA, USA) as previously described [10]. The *RBM20* variant p.Gly603Arg was introduced by the QuikChange^®^ Lightning Site-Directed Mutagenesis Kit (Agilent Technologies, Santa Clara, CA, USA) according to the manufacturer’s instructions. For the generation of the *RBM20* p.Glu792GlyfsTer9 the truncated fragment was amplified from the wildtype *RBM20* with the following primers (5′-GAATTCATGTCCCAGCCTCTCTTCAATC-3′; 5′-GGATCCGGTCCGGATGGGGGTGTCTGCTTTCCCCGCAGCCTGGC-3′) and inserted into pEYFP-N1 via *Eco*RI and *Bam*HI restriction sites. *RBM20*-encoding parts of all generated plasmids were verified by Sanger sequencing (Macrogen, Amsterdam, The Netherlands). 

### 2.7. Cell Culture and Transient Transfection

Cell culture and transfections were performed as previously described [10]. Briefly, C2C12 cells (ATCC, Manassas, VA, USA) were cultivated in Dulbecco’s Modified Eagle Medium supplemented with 10% fetal calf serum. Lipofectamine 2000 (ThermoFisher Scientific) was used for cell transfections according to the manufacturer’s instructions. Cells were plated 24 h before transfection on coverglasses coated with 0.02% collagen R-solution (SERVA electrophoresis, Heidelberg, Germany) and transfected with 750 ng of plasmid DNA in a 24-well plate at 70–90% confluence. Cells were fixed and embedded 48 h after transfection according to Gaertner et al. [10].

### 2.8. Immunohistochemistry

Frozen cardiac tissue was sliced into 5 µm sections and treated as previously described [10]. Anti-*RBM20* antibody (NBP1-91002, Novus Biologicals, Littleton, CO, USA) and Cy3-conjugated anti-rabbit IgG antibody (C2306, Sigma-Aldrich, St. Louis, MO, USA) were used for *RBM20* labelling. For nuclear staining, 4’,6-diamidine-2’-phenylindole dihydrochloride (DAPI) solution (Carl Roth, Karlsruhe, Germany) was used. Sections were embedded with Mowiol 4-88 (Carl Roth). 

### 2.9. Confocal Microscopy

Image acquisition was performed as previously described [10] with the TCS SP8 confocal microscope (Leica, Wetzlar, Germany). 

### 2.10. RNA-Sequencing

RNA-sequencing and read processing were performed as previously described [40]. Briefly, total RNA was isolated from about ~30 mg of myocardial tissue using the RNeasy Mini Kit (Qiagen, Hilden, Germany) and analyzed for RNA integrity number with the RNA 6000 Pico Kit (Agilent Technologies, Santa Clara, CA, USA). The TruSeq Stranded Total RNA Library Prep Kit with Ribo-Zero Gold (Illumina) was applied for RNA sample processing according to the manufacturer’s instructions. For final indexed libraries enrichment seven PCR cycles were performed and the indexed libraries were quantified with Qubit dsDNA HS assay kit (ThermoFisher Scientific) and qualified with Bioanalyzer using HS DNA Kit (Agilent Technologies). Equimolar amounts of each library were pooled and sequenced on Illumina HiSeq 3000 (single-end; 50 bp) using sequencing-by-synthesis chemistry v4, according to the manufacturer’s protocols and as previously described [40]. For each TruSeq RNA library, an average yield of 500 Mb of sequencing data with an average of 96% reads achieving a quality score ≥Q30 was produced. RNA-sequencing reads were mapped to the hg38 (GRCh38) reference genome sequence using STAR (v2.5.1b) [41]. To analyze *RBM20*tv-transcript frequency, BAM files were loaded into integrated genome viewer software (IGV_2.8.6, Broad Institute, Cambridge, MA, USA). At the corresponding *RBM20* genomic position, the number of transcripts with or without the variant was analyzed.

## 3. Results

### 3.1. A Combination of Truncating *RBM20* and TTN Variants Was Identified in a Patient with Severe DCM

We have identified a combination of truncating *RBM20* (NM_001134363) and *TTN* (NM_001267550) mutations in a German patient with a severe DCM (Figure 1). Besides both truncating mutations, the patient (IV.3) carries missense variants on each of the mutated alleles. He has inherited the *TTN* mutations p.Glu8271Gln (c.24811G > C) and p.Lys23669Ter (c.71005A > T) from his father (III.2, Figure 1) who also suffered from DCM. The *RBM20* mutations p.Gly603Arg (c.1807G > A) and p.Glu792GlyfsTer9 (c.2374dup) were inherited from the mother (III.3, Figure 1) who has arrhythmias but no cardiomyopathy. Due to the maternal and paternal inheritance pattern it is clear that the two *TTN* or *RBM20* variants are localized on the same allele. Two cousins of the patient (IV.8 and IV.9) were also diagnosed with an early onset DCM. One of the monozygotic twins was genotyped and was also carrier of the *TTN* mutations p.Glu8271Gln and p.Lys23669Ter. Cardiac tissue was available from three mutation carriers (III.2, IV.3, and IV.8, Figure 1).

The *RBM20* mutation p.Gly603Arg (rs558674954) has a minor allele frequency (MAF) of 0.00027 in GnomAD [42] and is classified as likely benign in ClinVar [43]. *RBM20* p.Glu792GlyfsTer9, which results in a frameshift and a subsequent premature termination codon is not listed in GnomAD. Although there are several reports of truncating *RBM20* mutations associated with cardiomyopathy [17,18,19,20,21,22,23], until now it is not completely understood how truncating variants contribute to the pathomechanism. As both *RBM20* variants are localized on the same allele, they have to be classified together. 

The *TTN* missense variant p.Glu8271Gln and the nonsense variant p.Lys23669Ter are both not listed in GnomAD [42]. Furthermore, the p.Lys23669Ter variant concerns a constitutively expressed *TTN* exon in the A-band (https://www.cardiodb.org/titin/index.php). It is known from the literature that truncating *TTN* mutations lead to a late onset cardiomyopathy in 95% of the cases [31]. According to the ACMG guidelines [35] the *TTN*-variant p.Lys23669Ter has to be classified as likely pathogenic (class 4). The missense variant p.Glu8271Gln has to be classified as variant of unknown significance as additional data for this variant are lacking. As both *TTN* variants are localized on the same allele, we have to classify them together. According to the ACMG guidelines [35], the *TTN* variant combination has to be classified as likely pathogenic (class 4).

### 3.2. The Ratio of RYR2- and TTN-Splice Variants Is Altered in the Patient with the *RBM20* Mutations

**Figure 2:**
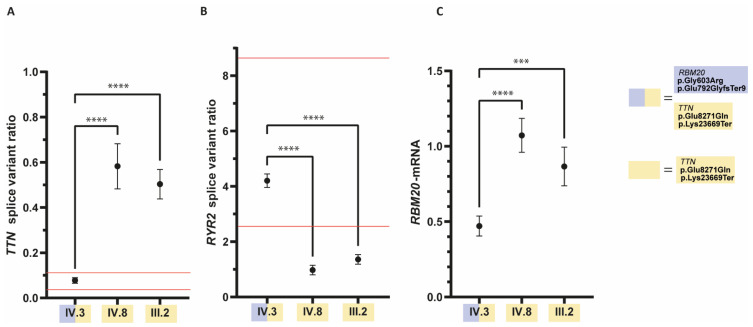
Quantitative real-time polymerase chain reaction (qRT-PCR) results of (**A**) *TTN*- (**B**) *RYR2*-splice variant ratios, and (**C**) relative quantification of *RBM20* mRNA. Data from affected family members with available cardiac tissue were measured as technical quintuplicates and are shown as means with standard deviation (SD). For statistical analyses one-way analysis of variance (ANOVA) with Dunnett’s multiple comparisons test was used. **** = *p* < 0.0001, *** = *p* = 0.0001. (**A**,**B**) The *TTN*- and *RYR2*-splicing in the index patient (IV.3) is significantly different from the splicing in his affected relatives, who are only carriers of the *TTN*-mutations. The *TTN*- and *RYR2*-splicing of IV.3 are within the previously defined reference range [10] for pathogenic *RBM20* mutations (red lines). (**C**) qRT-PCR analysis revealed decreased *RBM20* mRNA expression in the *RBM20* mutation carrier IV.3 in comparison to his relatives.

As previously shown, cardiomyopathy associated *RBM20* mutations lead to a missplicing of several cardiac genes, including *TTN* and *RYR2*. Recently, we have established a qRT-PCR-based splicing assay using explanted myocardial tissue to predict the pathogenic impact of *RBM20* mutations [10]. Analysis of the myocardial splicing of *RYR2* and *TTN* revealed aberrant splicing of *TTN* and *RYR2* in the index patient but not in his relatives carrying only the *TTN* mutations (Figure 2). The *TTN* splice variant ratio in the index patient was 0.08 ± 0.01 (Figure 2A) which is within the reference range for pathogenic *RBM20* mutations (0.03–0.11) as previously described [10]. The *TTN* splicing ratio of the index patient significantly differed (*p* < 0.0001; one-way analysis of variance (ANOVA) with Dunnett’s multiple comparisons test) from the *TTN* splicing ratio in his relatives who were not carriers of the *RBM20* mutation. The *TTN* splicing in IV.8 (0.58 ± 0.10) and III.2 (0.50 ± 0.07) (Figure 2A) was outside the reference range for pathogenic *RBM20* mutations [10]. 

Analysis of *RYR2* splice variant ratio (Figure 2B) revealed that the *RYR2* splicing ratio of IV.3 (4.21 ± 0.25) is within the previously defined reference range for pathogenic mutations (2.55–8.67) [10]. The *RYR2* splicing ratio in the index patient significantly differed (*p* < 0.0001; one-way ANOVA with Dunnett’s multiple comparisons test) from IV.8 (0.98 ± 0.17) and III.2 (1.36 ± 0.17) (Figure 2B) which were outside the reference range for pathogenic *RBM20* mutations [10]. 

In consequence we suggest that besides the likely pathogenic *TTN* mutations cosegregating with DCM in the family, the mutated *RBM20* allele identified in the patient leads to missplicing putatively contributing to the severe phenotype of the index patient IV.3. 

### 3.3. *RBM20*-p.Gly603Arg and -p.Glu792GlyfsTer9 Do Not Lead to an Abnormal Cytoplasmic Mislocalization

Recently, it was shown that the murine *Rbm20* and human *RBM20* mutants localized within the RS-domain of *RBM20* lead to a mislocalization of the protein in the cytoplasm [10,44,45].

**Figure 3:**
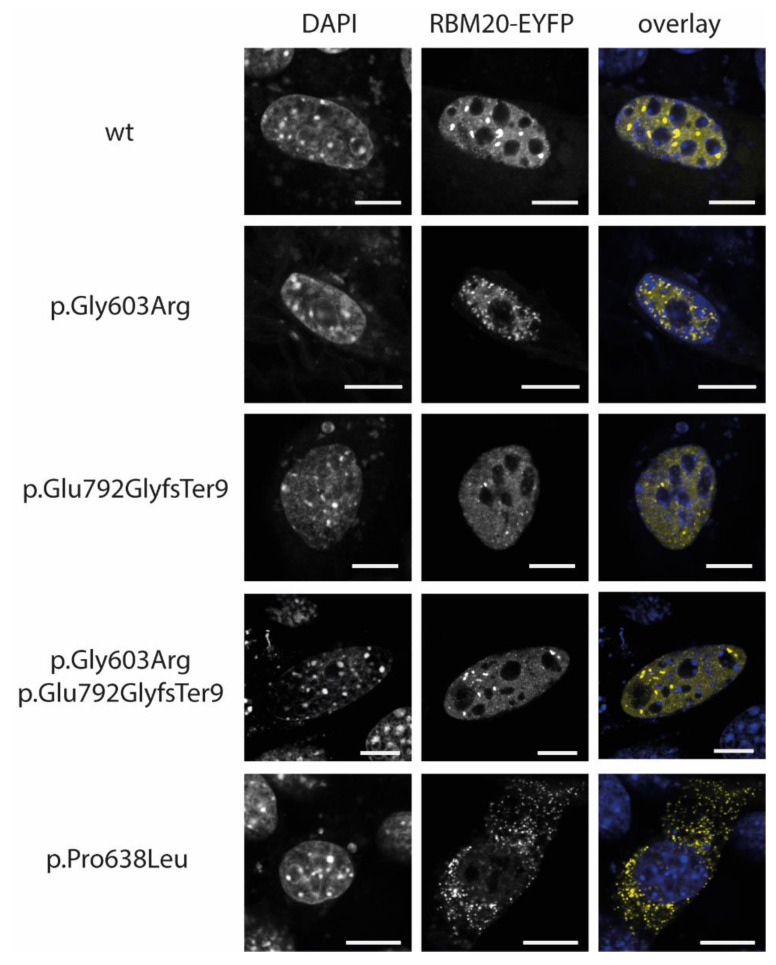
Localization analyses of RNA binding motif protein 20- enhanced yellow fluorescent protein (*RBM20*-EYFP) transfected C2C12 cells. *RBM20* is shown in yellow, and the nuclei were labelled with 4’,6-diamidine-2’-phenylindole dihydrochloride (DAPI, blue) in the overlay. EYFP tagged wildtype *RBM20* localizes in the nucleus. The mutant forms of *RBM20* (p.Gly603Arg and p.Glu792GlyfsTer9) are comparably localized in the nuclei. As described previously [10], only the mutant form *RBM20*-p.Pro638Leu shows an abnormal cytoplasmic localization. Scale bars = 10 µm.

**Figure 4:**
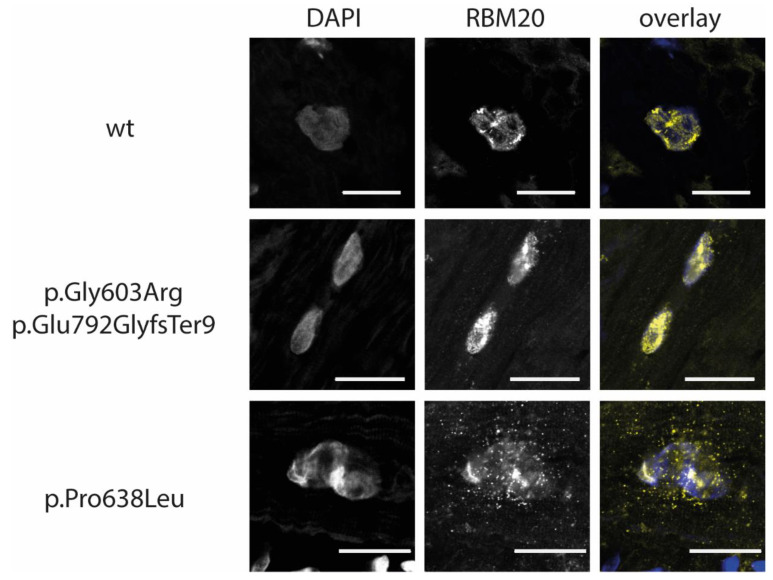
Immunohistochemical analysis of myocardial tissue of cardiomyopathy patients. Representative images of explanted myocardial tissue sections, which were labelled with primary anti *RBM20* and Cy3-conjugated secondary antibodies (yellow in the overlay), are shown. Nuclei were labelled with DAPI (blue in the overlay). Tissue from a DCM patient with no *RBM20* mutation was used as control. Comparable to the control sample *RBM20* is localized in the nuclei in explanted myocardial tissue from IV.3. Sections from a DCM patient with the pathogenic *RBM20* mutation p.Pro638Leu were used as a positive control for aberrant cytoplasmic *RBM20* localization. Scale bars = 20 µm.

In this study, we analyzed the localization of wildtype and mutant *RBM20*-enhanced yellow fluorescent protein (EYFP) fusion proteins in C2C12 cells and the localization of *RBM20* in the explanted tissue of the index patient. Wildtype *RBM20* protein localized predominantly in the nuclei (Figure 3). The p.Gly603Arg and p.Glu792GlyfsTer9 *RBM20*-EYFP proteins localized in the nuclei of C2C12 cells, which is in accordance with the localization of the mutations outside or C-terminal of the conserved RS-domain, as shown previously [10,44]. We analyzed the localization of *RBM20* in explanted myocardial tissue of the *RBM20*-mutation carrier IV.3 (Figure 4). Comparable to the cell culture experiments, mislocalization of *RBM20* in the cytoplasm, as it is observable for mutations in the RS-domain (p.Pro638Leu), was excluded. Therefore, it can be suggested that mislocalization of the *RBM20* mutations p.Gly603Arg and p.Glu792GlyfsTer9 in the cytoplasm is not part of the pathomechanism, leading to the missplicing observed in the index patient IV.3.

### 3.4. *RBM20*-p.Gly603Arg and -p.Glu792GlyfsTer9 Lead to *RBM20* Haploinsufficiency in the Index Patient

**Figure 5:**
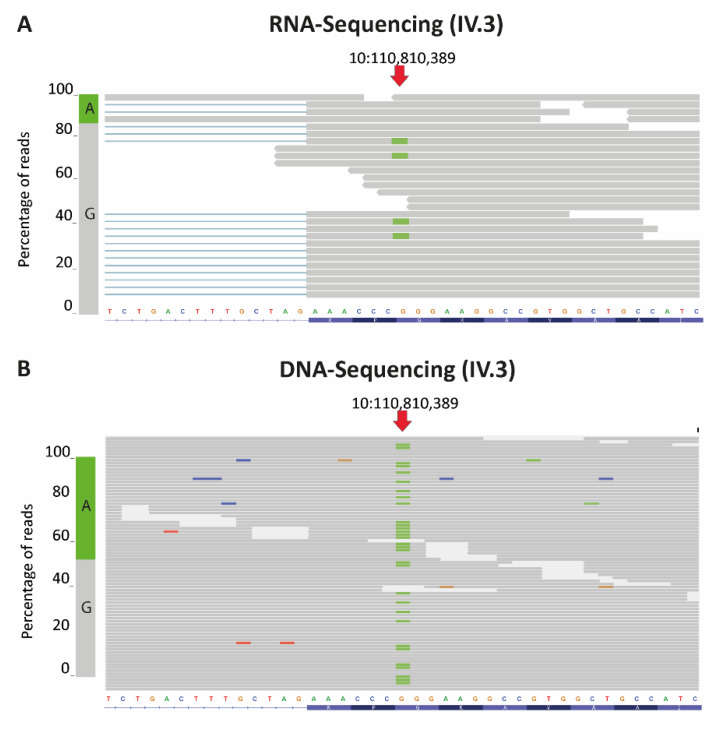
Integrated genome views of exon 8 of *RBM20*. The chromosomal position corresponding to *RBM20* c.1807 is marked with an arrow. Shown are the aligned reads obtained by RNA-sequencing (**A**) or DNA-sequencing (**B**) in the respective chromosomal region of index patient IV.3 (*RBM20* p.Gly603Arg+p.Glu792fsTer9 and *TTN* p.Glu8271Gln+ p.Lys23669Ter). At chromosomal position 10:110,810,389 approximately 15% of the RNA reads (**A**, bar on the left) represent the mutant form, whereas 46% of the reads (**B**, bar on the left) at chromosomal position 10:110,810,389 represent the mutant DNA form.

We performed RNA-sequencing analysis and revealed reduced levels of the *RBM20*- p.Gly603Arg and p.Glu792GlyfsTer9 allele in the index patient IV.3. However, we detected only expression of *RBM20*-p.Gly603Arg. However, as both mutations are localized on the same allele, the amount of p.Gly603Arg mRNA species (c.1807G > A exchange) is representative for the mutant allele. RNA-sequencing revealed that the mutant allele with an adenosine at position 1807 of *RBM20*-mRNA represents 15% of the mRNA species at this position (Figure 5A). Sequencing of genomic DNA (Figure 5B) proves that the observed allelic misdistribution is not just due to sequencing errors as the heterozygous mutant allele can be observed at a frequency of 46% in the genomic DNA of the patient.

qRT-PCR of *RBM20*-mRNA (Figure 2C) undermined the RNA-sequencing results as *RBM20*-mRNA content was reduced to a relative quantity (RQ) of 0.47 ± 0.07 in the *RBM20* mutation carrier compared with an RQ of 1.07 ± 0.11 and 0.87 ± 0.13 in his relatives who are carriers of the *TTN* mutations only. 

These results support the conclusion that the missplicing of *RBM20* target genes observed in the patient is caused by an *RBM20* haploinsufficiency. 

## 4. Discussion

Mutations in *RBM20* have been associated with DCM already in 2009 [9] and missplicing of several important cardiac genes like *TTN* was recognized as the potential pathomechanism induced by pathogenic *RBM20* mutations [8,13]. Nevertheless, it is likely that several different pathomechanisms might contribute to *RBM20*-dependent missplicing. It is well known that *RBM20* mutations in the highly conserved RS-domain lead to a cytoplasmic mislocalization of the *RBM20* protein [10,44,46,47]. Besides the cytoplasmic *RBM20* granula, which might contribute to the cardiomyopathy phenotype, the absence of the mutant protein from nuclei resulting in a functional *RBM20* haploinsufficiency leads to a missplicing in the patients. Currently, it is unclear if the abnormal cytoplasmic *RBM20* granula, absence of *RBM20* in the nuclei, or a combination of both is part of the underlying pathomechanism. 

Besides mutations in the highly conserved RS domain, mutations in the Glu-rich region were identified [10,16]. Of note, these mutations do not lead to a cytoplasmic mislocalization of *RBM20* but cause missplicing of its splicing targets.

From rodent models it is well known that heterozygous and homozygous *Rbm20* deficiency leads to a missplicing of *Rbm20* target genes [13,47]. In this manuscript, we show for the first time that human *RBM20* mutations might also lead to *RBM20* haploinsufficiency. qRT-PCR analysis reveals a reduction of *RBM20*-mRNA in the index patient compared to his relatives without any *RBM20* mutation. Based on RNA-sequencing data, it can be suggested that the expression of the mutant mRNA is decreased. It is well known that mRNAs carrying a PTC are frequently degraded by nonsense-mediated mRNA decay (NMD) [48,49,50]. Normally, NMD occurs if a premature termination codon (PTC) is located ≥50–55 nucleotides upstream of an exon-exon junction [51,52]. In *RBM20* p.Glu792GlyfsTer9, the insertion of a guanine in exon 9 leads to a frameshift and a PTC approximately 150 nucleotides upstream of the next exon-exon junction, which would be appropriate for the classical 3′ untranslated region exon junction complex dependent NMD. NMD is essential for the elimination of nonfunctional and/or toxic proteins which might result from a transcript with a PTC. Nevertheless, especially in proteins like *RBM20*, which act in a dose dependent manner [7,8], the cellular function of the protein might be dramatically disturbed by decreased protein levels. Interestingly, we demonstrate here that a decreased *RBM20* amount causes missplicing of *RBM20* target genes.

As haploinsufficieny can be confirmed for the mutant *RBM20* allele of the index patient and this haploinsufficiency obviously leads to missplicing of *RBM20* target genes, the ACMG criterion’s very strong evidence of pathogenicity (PVS1) is fulfilled [35,53]. Accordingly, the combination of *RBM20* mutations of the patient can be classified at least as likely pathogenic according to ACMG guidelines [35]. In addition, the pathogenicity of truncating *RBM20* mutations is underlined by the low ratio of observed/expected ratio (o/e) for loss of function mutations (pLoF) in the GnomAD [41]. Low o/e values are indicative for a low tolerance for loss of function mutations in one gene. For *RBM20*, the 90% confidence interval for pLoF is 0.09–0.29, which is below the recommended value of 0.35 for its classification as a loss of function variant [42]. 

It is estimated that truncating *TTN* mutations (*TTN*tv) are responsible for up to 25% of familial DCM cases [31,32,54,55]. *TTN*tv are especially enriched in A-band titin and affect constitutive exons in end-stage DCM cohorts [32]. The *TTN* mutation p.Lys23669Ter identified in the patients is localized in the constitutive exon 327, which encodes parts of the A-band titin. It is known that *TTN*tv commonly leads to a late onset cardiomyopathy with an average age for left ventricular diastolic dysfunction of 45 ± 14 in male patients [56]. The pathomechanisms of cardiomyopathy caused by *TTN*tv are not well understood. Based on work with hiPSC-CMs, sarcomere insufficiency was proposed as one of the contributors [57]. Ribosomal and RNA-sequencing revealed that *TTN* haploinsufficiency and NMD are not part of the pathomechanism in the hearts of *TTN*tv patients [32,58]. It is believed that additional stress (genetic or environmental) might be necessary for the development of DCM in titin insufficiency [59]. The pathogenic *RBM20* mutation combination identified in IV.3 is presumably an additional genetic factor contributing to the pathogenesis of DCM. In patients IV.8 and IV.9, environmental factors might be responsible for the early disease onset as it is well known that consumption of amphetamines might lead to the development of DCM [60,61,62,63].

Interestingly, the index patient’s mother (III.3), although carrying both *RBM20* mutations, shows no signs of DCM. This is in good accordance with previously published data, revealing a more severe disease expression in male *RBM20* mutation carriers [64]. Male carriers of pathogenic *RBM20* mutations show a significantly younger age and a lower ejection fraction at diagnosis than females [64]. Furthermore, the need for HTx is significantly higher in male mutation carriers [64]. 

## 5. Conclusions

In our manuscript we reveal that a combination of (likely) pathogenic mutations in *TTN* and *RBM20* leads to a severe cardiac phenotype. Furthermore our results imply that frameshift mutations in *RBM20* might lead to *RBM20* haploinsufficiency resulting in a splicing deficiency.

## Data Availability

The data that support the findings of this study are available from the corresponding author upon reasonable request.

## Supplementary Materials

The following are available online at https://www.mdpi.com/article/10.3390/genes12060883/s1, Table S1: Clinical baseline characteristics.
