# Supplementary material for: The Combined Human Genotype of Truncating TTN and RBM20 Mutations Is Associated with Severe and Early Onset of Dilated Cardiomyopathy
Source: Genes (Basel). 2021 Jun 8;12(6):883. doi: 10.3390/genes12060883 (PMC8228627; doi:10.3390/genes12060883)
Supplement: Supplementary file 1 [file genes-12-00883-s001.zip › genes-1232761-supplementary.pdf]

Table S1: Clinical baseline characteristics

**patient**	**sex**	**age [y]**	**EF**	**LVEDD**	**genotype**
III.2	m	41	20	82	*TTN* p.Glu8271Gln+p.Lys23669Ter
III.3	f	60	59	44	*RBM20* p.Gly603Arg+p.Glu792GlyfsTer9
IV.3	m	34	10	71	*TTN* p.Glu8271Gln+p.Lys23669Ter<br>*RBM20* p.Gly603Arg+p.Glu792GlyfsTer9
IV.8	m	23	15-20	68	*TTN* p.Glu8271Gln+p.Lys23669Ter
IV.9	m	23	35	n.a.	(*TTN* p.Glu8271Gln+p.Lys23669Ter)

Data were acquired either at the time of myocardial tissue sampling (left ventricular assist device implantation for III.2 and IV.8 or total artificial heartimplantation for IV.3) or at the time of the last available cardiologic examination (III.3 and IV.9). f=female, m=male, EF=ejection fraction in %, LVEDD=left ventricular end diastolic diameter in mm, n.a.=not available, wt=wildtype, y=years. The genotype of IV.9 is given in brackets as the patient is a monozygotic twin of IV.8 but was not genotyped.
